# Treatment Response of Gingival Squamous-Cell Carcinoma to Palliative Intent Immunotherapy

**DOI:** 10.3390/curroncol30120767

**Published:** 2023-12-18

**Authors:** Natalia Trehan, Angelina Debbas, Mykaihla Sternick, Jennifer Johnson, James C. Gates

**Affiliations:** 1 Department of Oral Medicine, Hospital of the University of Pennsylvania, Philadelphia, PA 19104, USA; 2 School of Dental Medicine, University of Pennsylvania, 240 South 40th Street, Philadelphia, PA 19104, USA; adebbas@upenn.edu; 3 Sidney Kimmel Medical College, Thomas Jefferson University, Philadelphia, PA 19107, USA; mykaihla.sternick@students.jefferson.edu; 4 Department of Medical Oncology, Department of Otolaryngology, Thomas Jefferson University, Philadelphia, PA 19107, USA; jennifer.johnson@jefferson.edu; 5 Oral and Maxillofacial Surgery, Hospital of the University of Pennsylvania, Philadelphia, PA 19104, USA

**Keywords:** immunotherapy, palliative intent, squamous-cell carcinomas, head and neck squamous-cell carcinomas

## Abstract

The use of PD-1 immune checkpoint inhibitor medications has become a common practice in the treatment of recurrent and metastatic head and neck squamous-cell carcinomas. Success in this setting has led to the investigation of their efficacy in locally advanced cases as a part of first-line therapy. In this report, we detail the treatment response to palliative intent immunotherapy of three geriatric patients with mandibular gingival squamous-cell carcinoma who decided against surgical intervention. Patient #1 was treated with pembrolizumab, a PD-1 inhibitor, and displayed complete clinical and radiologic response of the gingival mass after three months of treatment, which is ongoing at 19 months from initiation. Patients #2 and 3 are each on treatment with single-agent pembrolizumab, with partial response of their tumors, minimal side effects, and ongoing response at 9 and 5 months of treatment, respectively. Durable clinical treatment response to palliative immunotherapy, as is evident in this report, warrants further consideration and investigation in the geriatric population. With appropriate patient selection, surgery may be avoided and allow patients to prioritize quality of life over curative intent surgery.

## 1. Introduction

Curative intent treatment of head and neck squamous-cell carcinoma (HNSCC) is well established, based upon high levels of prospective evidence from consensus guidelines such as the National Comprehensive Cancer Network (NCCN) [1]. Treatment is often multimodal, which may include surgery, radiation, and chemotherapy. Palliative intent treatment aims to individualize care with attention to the risks and benefits of therapy. Given its efficacy and tolerability, immunotherapy may serve as a primary or adjunct palliative option in patients who are otherwise suboptimal candidates for surgery due to the risk/benefit ratio.

Immune checkpoint inhibitors (ICIs) such as anti-programmed cell death 1 (PD-1) receptor antibodies enhance the detection of tumor cells during immune-mediated destruction by T lymphocytes [2]. In the absence of such medication, PD-1 ligand (PD-L1) expressed on tumor cells binds to PD-1 expressed on mature cytotoxic T-cells, thereby inhibiting immune surveillance and destruction of tumor cells by these T-cells. This permits uncontrolled growth and differentiation of these tumor cells, as they evade normal T-cell surveillance. Anti-PD-1 ICIs remove the inhibitory mechanisms preventing tumor cell identification by T-cells and allow for their destruction. Several FDA-approved monoclonal antibodies targeting the PD-1/PD-L1 interaction are available. Pembrolizumab and nivolumab are approved for use in head and neck squamous-cell carcinoma, but have also demonstrated benefits in the treatment of several other tumor types, including melanoma, non-small-cell lung cancer, cutaneous squamous-cell cancer, Hodgkin’s lymphoma, and renal cell carcinoma [3]. Pembrolizumab also holds FDA indications for treatment of any solid tumor with microsatellite instability or a high tumor mutation burden. Identification of predictive biomarkers of efficacy has been challenging. Based upon the tumor type and drug, the use of PD-L1 immunohistochemistry scores is commonly applied. In HNSCC, this is performed through a combined positive score (CPS) [4].

FDA approval for the use of immune checkpoint inhibitors for head and neck cancer was obtained in 2018, based upon improved overall survival (OS) seen in two phase III clinical trials: CheckMate 141 (nivolumab) and KEYNOTE- 040 (pembrolizumab) [5,6]. These drugs were each compared to an investigator’s choice standard treatment of methotrexate, docetaxel, or cetuximab alone in patients with recurrent or metastatic disease who had already failed a first-line platinum-containing regimen. Later approval was given to pembrolizumab in first-line recurrent/metastatic therapy, either alone or in combination with chemotherapy, again due to an overall survival benefit. The determination of its use as a monotherapy or in combination with chemotherapy is partially based upon CPS scores, where increasing CPS score categories correlate to increased efficacy of PD-1 ICIs (<1, 1–19, >20) [4]. In addition to the improved efficacy noted with PD-1 ICIs, these agents exhibit a lower side effect profile than other chemotherapeutic agents, supported by a recent meta-analysis detailing that PD-1 inhibitors exhibited less fatigue, nausea, diarrhea, and fewer sensory neuropathies [7]. Reponses are durable for a subset of patients. Research is ongoing to predict which patients will derive the most clinical benefit.

Due to their success in the recurrent and metastatic setting, current trials and research aims to elucidate the efficacy of their use in the treatment of newly diagnosed, locally advanced disease. These include solo or combination therapy in the neoadjuvant and definitive concurrent settings with radiation or in the adjuvant setting. Immunotherapy is being explored for escalation of care in high-risk disease, de-escalation of care (avoidance of more toxic strategies) in lower-risk disease, and in the treatment of patients who are considered to be ineligible for certain modalities. Most frequently, immunotherapy is being explored as an alternative strategy for platinum-ineligible patients.

In this report, we detail the clinical and radiologic treatment response of a series of patients with gingival squamous-cell carcinoma to immune checkpoint inhibitors (ICIs). Each of these patients prioritized maintaining their quality of life and chose to avoid surgical intervention due to age and comorbid conditions. Immunotherapy provided maintenance of their quality of life with minimal side effects while providing ongoing treatment response.

## 2. Case Reports

### 2.1. Patient #1

**Figure 1:**
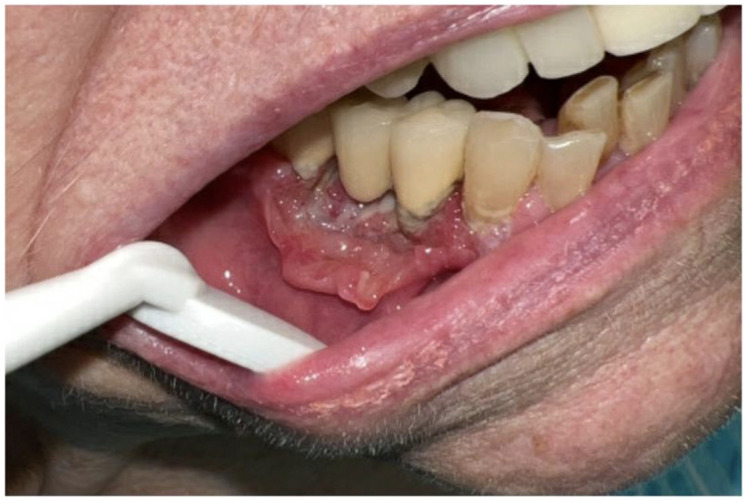
Pretreatment clinical photograph showing an exophytic, ulcerated gingival mass invading into the cortical bone of the right mandible.

**Figure 2:**
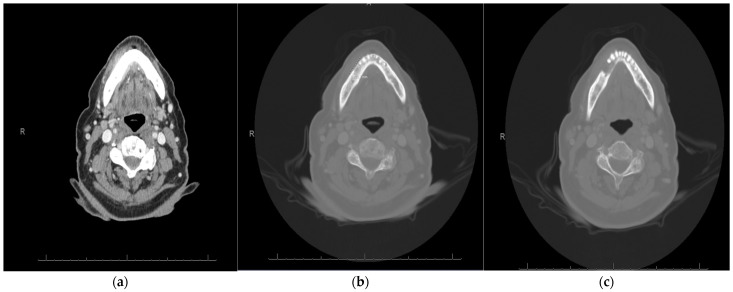
Pretreatment radiologic evaluation of a (**a**) soft tissue and (**b**) bone window of an axial slice computed tomography scan showing a 2 cm area of right buccal soft tissue thickening consistent with known tumor. In a subsequent (**c**) bone window, axial view, osteolysis and marrow invasion is noted adjacent to known carcinoma.

A 91-year-old female with minimal comorbidities presented for treatment of a gingival carcinoma. Upon examination, an approximately 3 cm × 2 cm exophytic, ulcerated, gingival mass was found invading into the cortical bone of the right mandible. A biopsy confirmed the presence of invasive squamous-cell carcinoma. There was clear clinical invasion of the mandibular alveolus in the vicinity of a prosthetic dental bridge (Figure 1). The patient did not display any cervical adenopathy, and her cranial nerve exam was normal. Laboratory evaluation revealed no signs of anemia or metastatic disease. Computed tomography imaging supported the diagnosis of carcinoma with mandibular invasion, past the cortex and into the marrow of the alveolus without evidence of regional or distal metastasis (Figure 2). Therefore, the patient was staged as a cT4aN0 squamous-cell carcinoma of the right mandible. We presented the patient with various treatment options, including curative surgical intervention that would require mandibular resection, reconstruction, selective neck dissection, and possible need for tracheostomy. Additionally, we discussed the possibility of curative intent, definitive, concurrent chemo-radiation. Though this is a less standard curative option with a lower potential cure rate, it was discussed as an option to avoid surgery. Finally, given the patient’s advanced age, we also discussed non-curative palliative treatment options, such as primary immunotherapy. We assessed the patient’s goals of care, and she emphasized the importance of maintaining quality of life, rather than quantity. She was referred to our geriatric oncology clinic for further discussion of goals of care and an assessment of life expectancy based upon age and comorbidity, aside from her oncologic prognosis. She also saw head and neck medical oncology, and she reiterated her goal of quality of life over long-term cure. Her CPS score was 85%.

**Figure 3:**
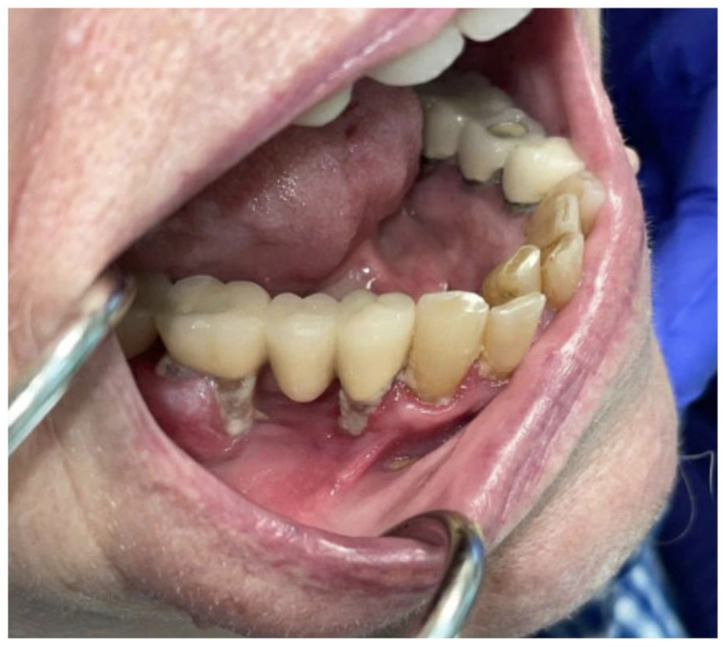
Post-treatment clinical photograph shows interval resolution of exophytic gingival mass with contracture of soft tissue and no discernible tumor or gingival irregularity after 3 months of treatment.

**Figure 4:**
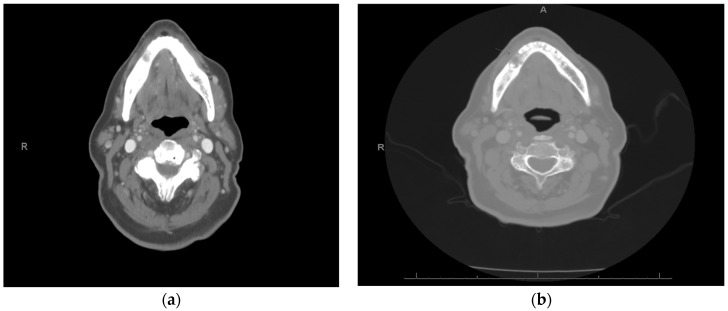
Restaging imaging (**a**) in soft tissue window of axial CT scan shows evidence of interval decrease in size of the right buccal thickening and symmetry with contralateral buccal region, while bone window (**b**) shows similar appearance of mandibular erosion performed approximately 3 months after treatment initiation.

After careful consideration, the patient and her family opted for primary palliative intent immunotherapy with a dose of 200 mg of pembrolizumab administered every 3 weeks. The treatment was well tolerated. After 3 months of treatment, clinical examination revealed regression of the exophytic mass and a transition to firm, pink, and firmly attached gingiva adherent to the underlying bone (Figure 3). Restaging imaging at the same time corroborated the clinical findings and showed complete resolution of the gingival mass (Figure 4). Given the patient’s favorable response to treatment, she continued therapy for 1 year. At that time, she continued to have a complete response by clinical assessments and near-complete reossification of the mandibular bony defect (Figure 4). A radiologic exam corroborated the clinical exam and she showed a complete response based on the RECIST criteria 1.1 [7]. A biopsy confirmed only inflamed squamous mucosa, and was negative for carcinoma and atypia. She discontinued treatment after 12 months of therapy and has been followed without any evidence of recurrence at the time of writing. She has remained off treatment with no evidence of recurrence and with ongoing efficacy at the time of this submission, 19 months post initiation of immunotherapy.

### 2.2. Patient #2

**Figure 5:**
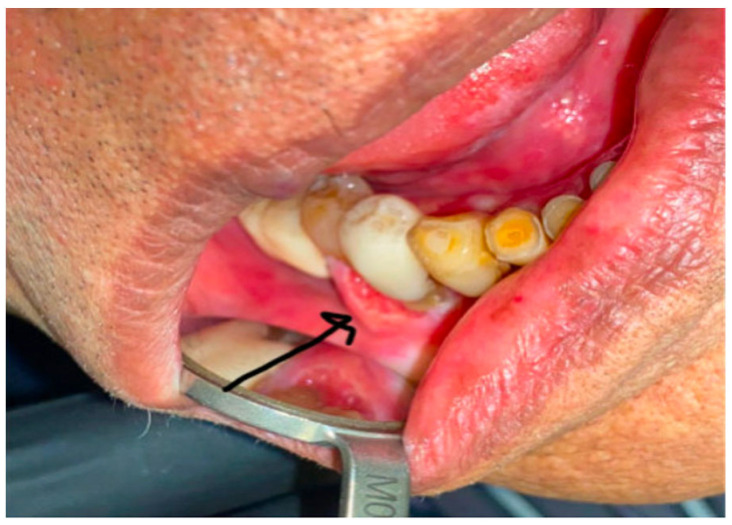
Pretreatment photograph of patient #2: arrow points to exophytic gingival mass between the two right mandibular premolars in the site of biopsy-proven squamous-cell carcinoma.

**Figure 6:**
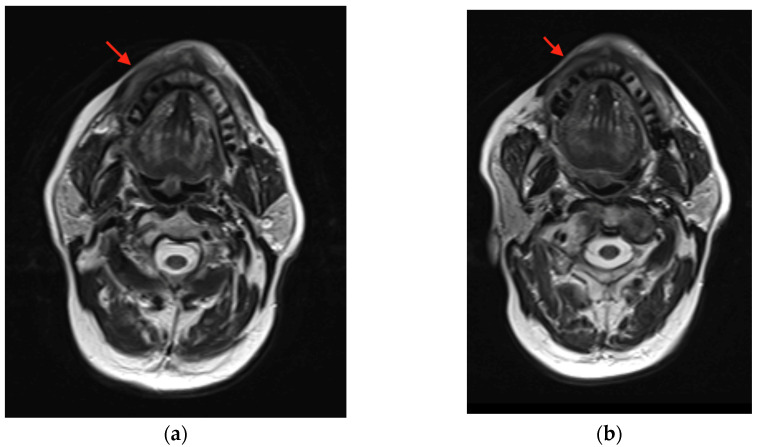
MRI shows (**a**) buccal thickening (red arrow) in site of carcinoma pretreatment and (**b**) interval decreased thickening (red arrow) of right buccal tissues post treatment.

An 82-year-old male with an ECOG classification of 0 presented for evaluation of a mild to moderately painful recurrent mandibular gingival carcinoma. He had previously been treated with a combination of surgery and radiation therapy for a cT2N0 squamous-cell carcinoma of the right mandible. Three years later, he presented with a recurrence in the right mandibular gingiva, specifically at the level of the premolars (Figure 5). A biopsy confirmed the recurrence as rT2N0 squamous-cell carcinoma. Pretreatment MRI revealed swelling in the right gingiva buccal region of the mandible (Figure 6a). Additionally, there was evidence of cortical erosion in the right mandibular premolar region, indicating superficial invasion of the recurrent malignancy. Notably, there was no cervical adenopathy.

**Figure 7:**
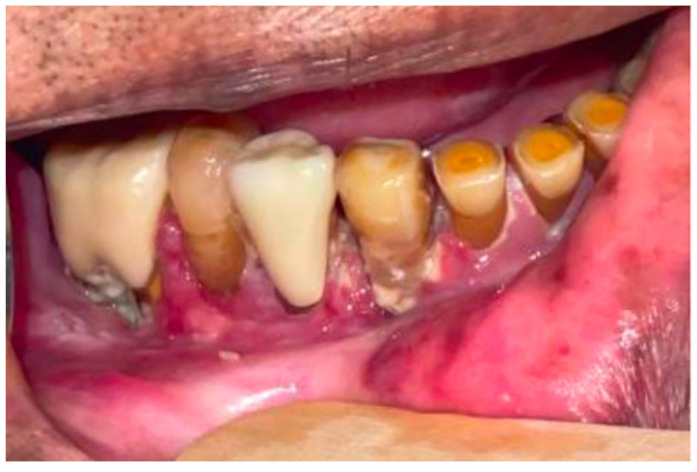
5 months post treatment of patient #2: clinical photograph shows resolution of the exophytic right mandibular tumor and normal contour of gingiva with associated erythema and pseudo membrane formation typical of inflammatory treatment change.

The patient was presented with treatment options, including curative surgical resection, which would have required mandibulectomy and a free fibula flap. Also, he was offered systemic chemotherapy and possibly reirradiation. However, the patient declined these options due to a desire to avoid mandibulectomy and further radiation treatment. Instead, the patient expressed interest in immunotherapy. His CPS score was found to be 30%, and he commenced treatment with pembrolizumab, administered at a dosage of 200 mg every 3 weeks. After three months of single-agent pembrolizumab, there was a noticeable decrease in the prominence of the tumor previously located around the right mandibular premolar region, and there was no longer evidence of exophytic growth (Figure 7). A post-treatment MRI was completed which showed a reduction in the swelling along the anterior right gingivobuccal region and stability of the erosion of the alveolar/periodontal region, particularly medial to the first and second premolars, near the site of the tumor (Figure 6b). Clinical examination findings thus demonstrated a partial treatment response. 

At the time of writing, the patient is 9 months post initiation of palliative intent pembrolizumab treatment for recurrent gingival squamous-cell carcinoma. He has shown a partial response to the treatment with minimal local site inflammatory side effects. He has no pain and thus treatment has improved his symptoms. He has fulfilled his ultimate treatment goals in preserving his overall quality of life and avoiding surgery.

### 2.3. Patient #3

**Figure 8:**
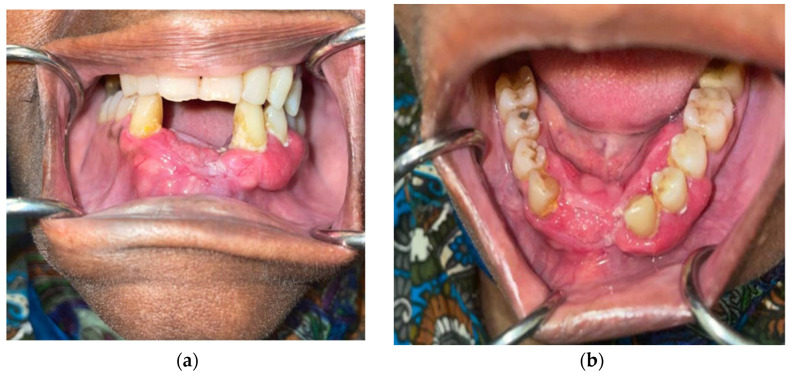
Pretreatment photographs of patient #3: (**a**) approximately 5 cm expansile, erythematous, gingival mass of the anterior and left mandible with (**b**) verrucous changes.

**Figure 9:**
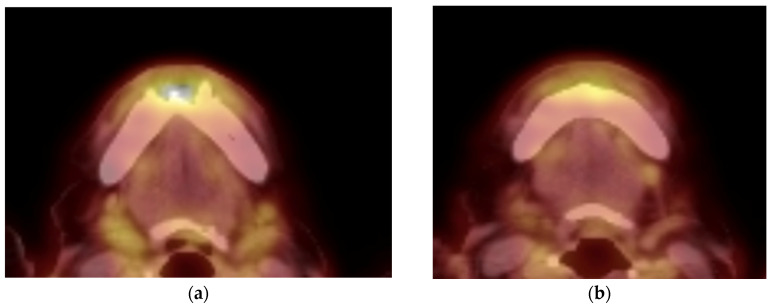
Radiologic examination of treatment effect of patient #3: (**a**) pretreatment and (**b**) post-treatment axial fused PET/CT image showing decreased avidity of the anterior mandible of SUV 5.9 to 4.4, respectively.

A 90-year-old female patient presented with an exophytic gingival mass in the anterior mandibular region at the site of prior dental extractions. A biopsy revealed moderately differentiated squamous-cell carcinoma (Figure 8). Contrast-enhanced CT of the neck, along with a PET/CT scan, were obtained and demonstrated erosion of the anterior cortex of the mandibular symphysis and left parasymphyseal region. Additionally, hypermetabolic left cervical lymph nodes involving lymph node stations 1–3 were present and presumed to be pathologic. Therefore, she was staged as a cT4N1 (Figure 9a).

A comprehensive treatment plan was presented to the patient, which included surgical intervention involving mandibulectomy, neck dissection, and the use of a free fibula flap. However, the patient and her family decided to decline this surgical option due to the patient’s advanced age, the extensive nature of the surgery and recovery required, and the subsequent recommendation for adjuvant radiation therapy.

An alternative treatment with primary chemotherapy and radiation therapy was discussed but also declined by the patient due to concerns of reduced survival as compared to surgery and the potential for increased toxicity from oral mucositis and pharyngeal dysphagia. 

**Figure 10:**
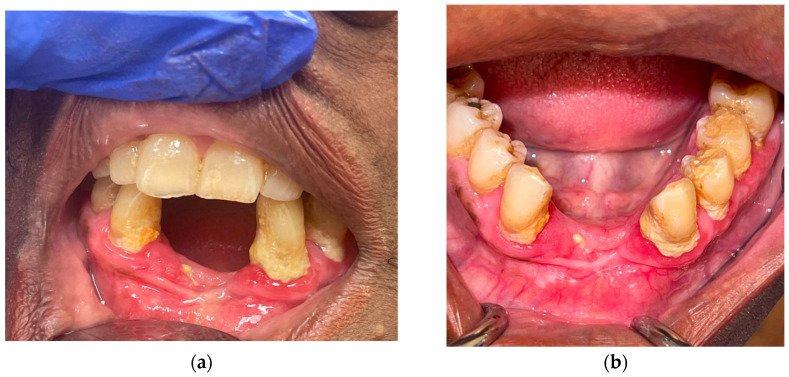
(**a**) Frontal and (**b**) occlusal post-treatment clinical photographs depicting contracture of the expansile mass and minimal verrucous epithelial change showing partial resolution of the tumor.

Palliative treatment options, including immunotherapy, were presented as a viable alternative. After some consideration, the patient began palliative immunotherapy with single-agent pembrolizumab. The standard infusion dosage of 200 mg every 3 weeks was initially administered at first. Once tolerance was established, the transition was made to extended dosing: 400 mg every 6 weeks. Treatment is ongoing at the time of this submission with a tentative plan for 24 months of therapy based on tolerance and efficacy. The patient has exhibited a partial response to immunotherapy. Clinically, we observed a decrease in erythema and swelling and partial resolution of the verrucous-appearing mass in and around the anterior mandibular teeth (Figure 10). After 1.5 months of treatment, a PET/CT scan was completed and revealed a partial metabolic response to the primary site (SUV uptake decreased from 5.9 to 4.4) as well as a complete response and resolution of avidity of left neck nodal metastases (Figure 9b). The patient has recently completed her third cycle of immunotherapy and has not reported any treatment-related side effects or adverse reactions. Furthermore, she expresses tolerability, satisfaction, and intention to continue treatment through to the end of the 24-month period of therapy. Her partial response and maintenance of her quality of life is ongoing (5 months) at the time of writing. 

## 3. Discussion

Palliative therapies for head and neck cancers are often patient-centered. In patients that are not suitable candidates for curative therapy, studies have shown palliative radiation therapy has the potential to mitigate symptoms secondary to cancer, at the risk of patients enduring harsh toxicities associated with this treatment [5]. Unlike radiation and cytotoxic chemotherapy, immunotherapy has the potential to produce durable treatment response with decreased side effects.

In this case series, we detail a complete and partial response of primary gingival squamous-cell carcinoma, and a partial response in a patient with recurrent mandibular squamous-cell carcinoma, to pembrolizumab monotherapy. All these patients had CPS scores greater than 20%, which afforded them the ability to avoid cytotoxic chemotherapy. Unfortunately, based upon the modest response rates seen in the KEYNOTE-048 clinical trial, we know that the majority of patients do not experience the positive treatment results that ours did. For example, in Keynote-48, the range of responses to single-agent pembrolizumab was from 4.5% in the CPS < 1 group to 13.5% in the CPS > 20 group [4]. Thus, more investigation is required to elucidate how to maximize response rates and to develop other biomarkers and tools to predict who will and who will not respond.

PD-L1 has shown promise as a predictive biomarker in cancer immunotherapy, which raises the question of whether the variability of response to immunotherapy is dependent on levels of PD-L1 expression in tumor cells. A meta-analysis which reviewed eight randomized control trials found a statistically significant reduction in the risk of death in patients who were PD-L1 positive and treated with PD-L1/PD-1 inhibition as monotherapy when compared to control groups (HR = 0.66, 95% confidence interval 0.59 to 0.74); however, patients who were PD-L1 negative and treated with PD-L1/PD-1 inhibitors were also associated with better survival compared to controls (HR = 0.80, 95% confidence interval 0.71 to 0.90). In six of the eight trials, control group patients were treated with conventional chemotherapy, while the remaining two trials used control groups treated with other immunotherapies which did not act directly on the PD-1 pathway [8]. These findings support the use of PD-L1 expression as one part of a multifactorial clinical picture when predicting responses to immunotherapy targeting PD-1/PD-L1. Another possible predictor of immunotherapy susceptibility is deficiency in mismatch repair machinery with high microsatellite instability (dMMR/MSI-H) [9]. In the three cases presented, dMMR/MSI-H was not monitored, as it is not routinely done so in the care of these patient groups.

The presence of prior treatment modalities in the patients presented in this series prior to the initiation of immunotherapy had an indeterminate effect on their responses to pembrolizumab. Patient 1 was naïve to oncologic treatments and had the most favorable treatment response, with complete response by clinical assessment, while patient 3, who also presented for initial treatment, demonstrated a partial response, but to more advanced disease which included regional metastasis. Patient 2 demonstrated a partial response yet is being treated for recurrence after undergoing prior combination therapy of surgery and radiation. This continues to be an area of great focus in current clinical trials regarding the optimal timing for immunotherapy. As clinical and preclinical research progresses, immune checkpoint inhibitors (ICIs) may be employed earlier and in conjunction with standard treatments. Recent preclinical research presents evidence that preservation of intact lymphatics enhances the antitumor effects of ICI immediately following treatment. Saddawi et al. compared two murine mouse models afflicted with head and neck squamous-cell carcinoma. They found that those treated with standard regional lymph node ablation (neck dissection and radiation) have diminished ICI responses and worse overall survival as compared to treatment-naïve group. Moreover, they noted an upregulation of type I dendritic cells and type I interferon signaling in the treatment-naïve group, which are necessary for ICI response and are lost in the group that receives lymph node dissection and radiation. While additional future research and clinical trials are needed to test and corroborate these findings in humans, their work provides insight into the mechanisms by which ICIs achieve their response and how standard therapies may remove these innate immunologic responses necessary for robust ICI response. Further research and developments could shift ICI use further into primary treatment and continue to change the paradigm of HNSCC treatment [10].

Important to the consideration of oncologic treatment in the geriatric population is that immunotherapy has been documented to have a lower incidence and severity of side effects as compared to other treatment [5]. A case report studying a patient with primary squamous-cell carcinoma compares the plethora of adverse effects that the patient endured while undergoing chemotherapy as compared to the improved quality of life while using a concurrent approach of radiation therapy and pembrolizumab [11]. This patient initially underwent intensity-modulated radiation therapy, with 7 weeks of concurrent carboplatin–paclitaxel administered weekly. They experienced grade 2 fatigue, mucositis, and dermatitis resulting in desquamation, as well as grade 3 dysphagia and dry mouth accompanied by thickened and excessive secretions. Long-term, they developed chronic xerostomia and dysphagia, resulting in the placement of a permanent gastrostomy tube. The patient then opted to undergo palliative treatment with pembrolizumab for pulmonary metastatic disease due to the adverse effects. They demonstrated decreased size of metastatic pulmonary nodules while experiencing reduced side effects [11].

In Natesan’s 2023 abstract, at their expansive cancer center network of sites in New York, Pennsylvania, and Ohio, the frequency of pembrolizumab dosing every six weeks exhibited a notable increase at the clinical sites compared to non-clinical sites. Additionally, there was a significant prevalence of transitioning from a three-week to a six-week dosing schedule at the clinical sites [12]. It is imperative to undertake further investigation to elucidate the underlying patient-, provider-, and institutional-level factors contributing to the variations in real-world pembrolizumab dosing practices. Moreover, exploring the repercussions of these dosing patterns on care delivery outcomes, including cost and accessibility, necessitates comprehensive examination.

Recent advancements in the treatment of head and neck squamous-cell carcinoma using ICIs have significantly impacted the established treatment paradigms. Initially, immunotherapy was reserved for use in the recurrent and metastatic settings as a second-line therapy. It is now standard of care as first-line therapy for recurrent/metastatic disease. Given its efficacy and decreased side effect profile compared to cytotoxic chemotherapy, its use is being expanded into virtually all aspects of head and neck oncologic care. Geriatric head and neck oncology patients present a unique challenge regarding life expectancy, ability to tolerate treatment, and the desire to maintain their quality of life [13,14]. For these reasons, primary palliative intent immune checkpoint inhibition was used for our three patients. This report highlights the potential for durable treatment response with a lower side effect profile, improvement in symptoms, and maintained quality of life. Based on these observations, we propose the use of primary palliative intent immune checkpoint inhibition for select geriatric oncology patients either as monotherapy or in combination based upon CPS score in those wishing to avoid the morbidity and toxicity of curative intent treatment.

## 4. Conclusions

The use of immune checkpoint inhibitors (ICIs), particularly anti-PD-1 antibodies like pembrolizumab and nivolumab, has revolutionized the treatment landscape for head and neck squamous-cell carcinoma (HNSCC). These therapies, initially reserved for recurrent and metastatic cases, have now become a standard of care, offering a remarkable balance between efficacy and reduced side effects when compared to traditional cytotoxic chemotherapy. The case series discussed in this report underscores the potential for durable treatment response and symptom improvement in select geriatric patients who prioritize maintaining their quality of life while avoiding the morbidities associated with curative intent treatment. As immunotherapy continues to evolve and expand into various facets of HNSCC treatment, it provides a patient-centered, palliative alternative for those who may not be ideal candidates for surgery or aggressive treatments. The ongoing research in this field aims to further refine patient selection criteria and predictive biomarkers, ultimately improving the chances of a positive treatment response. Immune checkpoint inhibition, whether as monotherapy or in combination based on CPS score, presents a promising approach for oncology patients seeking to optimize their care and enhance their quality of life.

## Data Availability

The data presented in this study are available in this article.
